# Treatment of ankylosing spondylitis with TNFα inhibitors does not affect serum levels of tryptophan metabolites

**DOI:** 10.1007/s10787-023-01317-7

**Published:** 2023-08-30

**Authors:** Joanna Witoszyńska-Sobkowiak, Dorota Sikorska, Rafał Rutkowski, Karolina Niklas, Iwona Żychowska, Włodzimierz Samborski

**Affiliations:** 1 Department and Clinic of Rheumatology, Rehabilitation and Internal Diseases, Poznan University of Medical Sciences, 28 Czerwca 1956 Roku 135/147, 61-545 Poznan, Poland; 2 Department of Pathophysiology, Poznan University of Medical Sciences, Poznan, Poland

**Keywords:** Tryptophan, Serotonin, Kynurenine, Quinolinic acid, Ankylosing spondylitis, TNFα inhibitors

## Abstract

The imbalance between the kynurenine and serotonin pathways can have serious consequences, e.g., depression. One of the factors leading to the imbalance between the pathways of tryptophan metabolism is inflammation. The aim of our study was to assess the impact of treatment with tumor necrosis factor-alpha (TNFα)-inhibitors on tryptophan metabolism in patients with ankylosing spondylitis (AS). Forty patients with AS (twenty-eight males, twelve females; mean age 40 ± 11 years), qualified to receive anti-TNF-α treatment, were prospectively assessed. As a control group, 20 healthy volunteers (7 males and 13 females, mean age 38 ± 5 years) were recruited from the general population. Patients underwent full clinical and biochemical assessment before and after 6 months of therapy. Disease activity was assessed by the Bath Ankylosing Spondylitis Disease Activity Index (BASDAI). The presence of depressive disorders was assessed with Beck's Depression Inventory (BDI) scale. Serum concentrations of tryptophan, serotonin, kynurenine, and quinolinic acid were measured. The predominance of the kynurenine pathway in AS patients (compared to the control group) was demonstrated (*p* < 0.001). Surprisingly, no significant changes in serum levels of tryptophan and its metabolites in AS patients after treatment were found, despite clinical improvement. Moreover, the components of tryptophan metabolism did not correlate significantly with the clinical activity of AS, depression nor laboratory inflammatory markers. Probably some other factors influence the pathways of tryptophan metabolism in patients with ankylosing spondylitis.

## Introduction

Tryptophan is an essential amino acid, involved in several physiological processes including neuronal function, immunity, and gut homeostasis (Comai et al. 2020). In humans, tryptophan is metabolized by two pathways: the kynurenine and the serotonin pathways, resulting in formation of kynurenine and kynurenine metabolites (like kynurenic acid and its antagonist quinolinic acid), serotonin and melatonin. All of these metabolites play important roles. Serotonin and melatonin regulate neurotransmission, cognitive functions, and influence circadian rhythm. Kynurenines serve important signaling functions in organ communication and modulate endogenous inflammation. Although the major catabolic pathway of tryptophan is the kynurenine pathway (which accounts for 95% of dietary tryptophan degradation), the balance between the two ways is very important (Comai et al. 2020; Kanova and Kohout 2021; Wang et al. 2015).

The imbalance between the kynurenine and serotonin pathways can have serious consequences, e.g., depression. For all that, little is known about the mechanisms maintaining the balance between them (Comai et al. 2020; Li et al. 2017; Bryleva and Brundin 2017). One of the factors leading to the imbalance between the two pathways of tryptophan metabolism is inflammation (Comai et al. 2020; Kanova and Kohout 2021; Wang et al. 2015). It has been hypothesized that induction of the kynurenine pathway by inflammation may reduce the availability of tryptophan and consequently lead to reduced serotonin synthesis (Cervenka et al. 2017). Additionally, growing evidence suggests that inflammation is associated with a dysregulation of the kynurenine pathway, causing an increase in the concentration of neurotoxic products such as quinolinic acid (Comai et al. 2020; Kanova and Kohout 2021; Wang et al. 2015).

Chronic inflammation in autoimmune diseases probably affects the tryptophan metabolic pathways—that may be one of the reasons for the high incidence of depression in this group of patients. However, the exact role of tryptophan pathways in specific rheumatic diseases has not yet been fully understood (Ogbechi et al. 2020; Buras et al. 2016; Ren et al. 2023). It seems that effective treatment of rheumatic diseases, and in particular with the use of new biological drugs—inhibitors of pro-inflammatory cytokines, should restore the balance between the kynurenine and serotonin pathways. However, the exact effect of biologic disease-modifying antirheumatic drugs on changes in tryptophan metabolism is also unknown (Rani et al. 2022).

Our particular attention was drawn to ankylosing spondylitis (AS), which is characterized by chronic inflammation and frequent occurrence of depressive disorders, but probably there is no direct link between C-reactive protein (CRP)-mediated inflammation and depressive symptoms (Omar et al. 2022; Webers et al. 2020). Also, the importance of tryptophan metabolism pathways in ankylosing arthritis is not clearly understood (Huang et al. 2022). Even less is known about the effect of biologic therapy in ankylosing spondylitis on the course of depression and the pathways of tryptophan metabolism (Rani et al. 2022). Already in 1964, attention was paid to changes in tryptophan metabolism in rheumatoid arthritis, although no such changes have been demonstrated in ankylosing spondylitis (Beetham et al. 1964). In more recent study, altered kynurenine pathway metabolism was found in patients with ankylosing spondylitis and effective therapy with tumor necrosis factor-alpha (TNFα) inhibitors resulted in a decrease in kynurenine/tryptophan ratio, even though the effect of treatment on other metabolites appears to be limited (Eryavuz Onmaz et al. 2021). Other authors have shown that serotonin levels are low in patients with ankylosing spondylitis and decrease even further during biological treatment (Klavdianou et al. 2016). Therefore, the results are not unambiguous and require further analysis.

The aim of our study was to assess the impact of biologic treatment with TNFα-inhibitors on tryptophan metabolism in patients with ankylosing spondylitis. The relationship between the disease activity, inflammatory markers, symptoms of depression, and products of tryptophan metabolism was also assessed.

## Materials and methods

### Patients

Forty consecutive Caucasian patients with ankylosing spondylitis (twenty-eight males, twelve females; mean age 40 ± 11 years) qualified to receive anti-TNF-α treatment were prospectively assessed before and after 6 months of therapy.

The inclusion criteria were as follows: (1) the diagnosis of ankylosing spondylitis according to the American-European Consensus Group classification criteria (Rudwaleit et al. 2009), (2) high disease activity and the unsuccessful treatment for AS with two types of non-steroidal anti-inflammatory drugs (NSAIDs) according to the current guidelines (van der Heijde et al. 2017). Exclusion criteria were (1) heart failure (NYHA class ≥ II), (2) respiratory, kidney or liver failure, (3) an acute or opportunistic infection in the last 3 months, (4) a documented HIV infection, (5) cancer (including a disease identified and cured in the past 5 years), (6) demyelinating diseases, (7) pregnancy, (8) age under 18 or over 65, (9) a diagnosis of a mental disorder, including depression, and/or a history of psychiatric treatment.

All the patients received anti-TNFα treatment (adalimumab, certolizumab, etanercept, golimumab or infliximab) as per standard protocols (Rudwaleit et al. 2009), without any changes in treatment throughout the observation period. In patients with severe symptoms, NSAIDs were ordered at the discretion of allowed, but such patients were included in the analysis only when these drugs were administered at the same doses for at least 4 weeks before and during the whole anti-TNFα therapy.

As a control group, 20 healthy volunteers (7 males and 13 females, mean age 38 ± 5 years) were recruited from the general population. The participants in the control group did not have any chronic diseases (was not taking any medications) or depression. Unfortunately, the gender distribution in the control group was slightly different than in the study group, which is one of the additional limitations of our research.

### Clinical assessment of disease activity

Patients underwent full clinical and biochemical assessment before and after 6 months of anti-TNFα therapy. Disease activity was assessed by the Bath Ankylosing Spondylitis Disease Activity Index (BASDAI) (Zochling 2011). The presence of depressive disorders was assessed with Beck's Depression Inventory (BDI) scale (Robinson and Kelley 1996).

### Laboratory analysis

Blood samples were collected in a fasting state at the time of clinical examination. Serum obtained was aliquoted and stored at −80 °C until measured. Serum concentrations of tryptophan, serotonin, kynurenine, and quinolinic acid were measured with specific immunoassays (Immundiagnostik, Bensheim, Germany), as per manufacturer’s instructions. Estimated detection levels were 1.46 µmol/l, 6.9 ng/ml, 0.12 µmol/l, and 1.23 ng/ml for tryptophan, serotonin, kynurenine, and quinolinic acid, respectively. All other laboratory tests were performed routinely by the hospital central laboratory.

### Statistical analysis

Statistical analyses were performed with the Statistica 13.0 software (StatSoft Polska, Kraków, Poland). Normality of the data distribution was tested with the Shapiro–Wilk test. As most of the data obtained (except age and BMI) did not consistently display a normal distribution, and due to the small size of the analyzed groups, the data were analyzed with nonparametric statistics. The Wilcoxon test was used to compare related parameters before and after treatment. Differences between unpaired data were analyzed with the Mann–Whitney test or *χ*^2^ test, respectively. Correlations between variables were analyzed with the Spearman’s rank correlation coefficient. The data were presented as medians and interquartile ranges or means and standard deviations or percentage, as appropriate. Differences were considered significant at *p* < 0.05.

## Results

### Characteristics of the study groups

Detailed characteristics of the study groups are presented in Table 1.

**Table 1:** Characteristics of the study and control group

	Ankylosing spondylitis (*n* = 40)	Healthy control (*n* = 20)	*p* value
Sex	28 males and 12 females	7 males and 13 females	0.010
Age (years)	40 ± 11	38 ± 5	0.486
Duration of the disease (years)	10 ± 7	0	< 0.001
BMI (kg/m^2^)	25 ± 4	24 ± 3	0.684
Comorbidities	Hypertension: *n* = 6 Hypothyroidism (in the euthyroid stage): *n* = 2	0	< 0.001
Type of TNFα inhibitor	Adalimumab: *n* = 25 Certolizumab pegol: * n* = 10 Etanercept: * n* = 1 Golimumab: * n* = 1 Infliximab: * n* = 3	0	< 0.001

Data presented as mean ± standard deviation. *p* value calculated with the Mann–Whitney test or *χ*^2^ test

*BMI* body mass index, *TNFα* tumor necrosis factor-alpha

### Response to treatment

Six months of biologic treatment with TNFα inhibitors resulted in a significant improvement—the favorable response was reflected both by clinical and biochemical criteria—Table 2.

**Table 2:** Clinical and biochemical response to 6-month treatment

	Before treatment (*n* = 40)	After treatment (*n* = 40)	*p* value
BASDAI	7.0 (5.6–7.6)	1.7 (1.1–2.5)	< 0.001
VAS (mm)	75.5 (68.0–81.5)	17.5 (10.0–30.0)	< 0.001
ESR (mm/h)	14.0 (8.0–23.0)	4.0 (2.0–7.0)	< 0.001
hsCRP (mg/dl)	7.7 (2.1–13.7)	1.1 (0.5–3.8)	< 0.001
WBC (10^9^/l)	8.0 (6.1–9.4)	7.1 (5.8–8.2)	0.005
Neutrophils (10^9^/l)	4.8 (3.6–6.0)	3.7 (2.5–4.8)	< 0.001
Lymphocytes (10^9^/l)	2.0 (1.8–2.2)	2.4 (2.2–2.9)	< 0.001
N/L ratio	2.4 (2.0–2.9)	1.5 (1.1–1.9)	< 0.001
RBC (10^12^/l)	4.7 (4.4–5.1)	4.9 (4.6–5.0)	0.032
Hgb (g/dl)	14.0 (12.5–15.0)	14.4 (13.6–15.3)	< 0.001
PLT (10^9^/l)	292.5 (236.0–325.0)	254.0 (216.0–301.0)	0.002

Data presented as median and interquartile range. *p* value calculated with the Wilcoxon test

*BASDAI* Bath Ankylosing Spondylitis Disease Activity Index, *VAS* visual analog scale, *ESR* erythrocyte sedimentation rate, *hsCRP* high sensitivity C-reactive protein, *WBC* white blood cells, *N/L ratio* neutrophils to lymphocytes ratio, *RBC* red blood cells, *Hgb* hemoglobin, *PLT* platelets

### Tryptophan metabolism

The obtained results indicate altered tryptophan pathway metabolism with the predominance of the kynurenine pathway in patients with ankylosing spondylitis compared to the control group —Table 3﻿.

Surprisingly, the treatment did not significantly affect tryptophan metabolism —Table 4.

**Table 3:** Tryptophan metabolism in the ankylosing spondylitis group and the control group

	Ankylosing spondylitis (*n* = 40)	Control (*n* = 20)	*p* value
Tryptophan before treatment (µmol/l)	167 (142–205)	40 (35–44)	< 0.001
Tryptophan after treatment (µmol/l)	167 (144–190)	40 (35–44)	< 0.001
QA before treatment (ng/ml)	3.2 (2.9–3.6)	1.1 (0.8–1.9)	< 0.001
QA after treatment (ng/ml)	3.1 (2.8–3.6)	1.1 (0.8–1.9)	< 0.001
Kynurenine before treatment (µmol/l)	2.5 (2.0–2.8)	1.3 (1.1–1.7)	< 0.001
Kynurenine after treatment (µmol/l)	2.3 (1.9–2.8)	1.3 (1.1–1.7)	< 0.001
Serotonin before treatment (ng/ml)	27 (1–75)	217 (167–326)	< 0.001
Serotonin after treatment (ng/ml)	25 (3–43)	217 (167–326)	< 0.001

Data presented as median (IQR). *p* value calculated with the Mann–Whitney *U* test or Wilcoxon test

*QA* quinolinic acid

Moreover, the components of tryptophan metabolism did not correlate significantly with the clinical activity of the ankylosing spondylitis nor laboratory markers of inflammation.

**Table 4:** Tryptophan metabolism before and after treatment

	Before treatment (*n* = 40)	After treatment (*n* = 40)	*p* value
Tryptophan (µmol/l)	167.3 (142.8–204.9)	167.0 (144.4–189.6)	0.104
Serotonin (ng/ml)	27.3 (1.4–74.6)	25.3 (3.2–43.2)	0.338
Kynurenine (µmol/l)	2.5 (2.0–2.8)	2.3 (1.9–2.8)	0.143
Quinolinic acid (ng/ml)	3.2 (2.9–3.6)	3.1 (2.8–3.6)	0.340
Serotonin/tryptophan ratio	0.16 (0.01–0.44)	0.14 (0.01–0.26)	0.671
Kynurenine/tryptophan ratio	0.01 (0.01–0.02)	0.01 (0.01–0.02)	0.150
Serotonin/kynurenine ratio	8.27 (0.78–28.07)	11.36 (1.04–24.51)	0.530

Data presented as median and interquartile range. *p* value calculated with Wilcoxon test

### Depression

Considering cutoff scores of ≥ 11 for BDI, before treatment, 14 of the 40 participants (35%) had depression. Statistically significant improvement in the BDI score was observed after treatment (10 ± 6 vs. 5 ± 4; *p* < 0.001) and only five patients (12%) achieved the BDI ≥ 11 after treatment. Surprisingly, the groups of patients with and without depression did not differ significantly in terms of disease activity, inflammatory parameters, or tryptophan metabolites—Table 5. Additionally, the level of depression did not significantly correlate with these variables (Table 5).

**Table 5:** Comparison of patients with and without depression

	Before treatment			After treatment		
	BDI ≥ 11 (*n* = 14)	BDI < 11 (*n* = 26)	*p* value	BDI ≥ 11 (*n* = 5)	BDI < 11 (*n* = 35)	*p* value
BASDAI	6.5 (5.3–7.2)	7.2 (5.8–7.6)	0.292	2.6 (2.0–2.7)	1.6 (1.0–2.4)	0.219
VAS (mm)	72.5 (65.0–85.0)	76.5 (70.0–81.0)	0.922	30.0 (17.0–37.0)	16.0 (10.0–30.0)	0.295
ESR (mm/h)	16.0 (4.0–18.0)	13.0 (8.0–24.0)	0.664	6.0 (4.0–10.0)	4.0 (2.0–6.0)	0.451
hsCRP (mg/dl)	5.2 (0.9–8.0)	7.9 (2.2–10.0)	0.279	9.6 (0.0–10.0)	1.0 (0.4–3.1)	0.486
WBC (10^9^/l)	7.9 (6.2–8.8)	8.0 (6.0–9.5)	0.528	7.6 (6.0–8.2)	7.1 (5.7–8.1)	0.637
Neutrophils (10^9^/l)	5.0 (3.6–5.5)	4.7 (3.6–6.2)	0.769	4.5 (3.8–4.6)	3.6 (2.5–4.9)	0.367
Lymphocytes (10^9^/l)	1.9 (1.7–2.2)	2.0 (1.8–2.2)	0.598	2.2 (1.9–2.7)	2.4 (2.2–2.9)	0.285
N/L ratio	2.4 (1.7–3.2)	2.4 (1.9–2.9)	0.776	2.0 (1.7–2.4)	1.4 (1.4–1.9)	0.110
RBC (10^12^/l)	5.0 (4.8–5.3)	4.6 (4.3–5.0)	0.011	4.9 (4.9–5.0)	4.8 (4.5–5.0)	0.326
Hgb (g/dl)	14.9 (14.0–15.7)	12.9 (12.4–14.6)	0.010	15.2 (14.2–15.3)	14.4 (13.6–15.3)	0.652
PLT (10^9^/l)	269.0 (218.0–327.0)	298.5 (251.0–323.0)	0.364	241.0 (217.0–348.0)	257.0 (215.0–295.0)	0.759
Tryptophan (µmol/l)	199.1 (160.8–236.5)	159.2 (141.6–189.5)	0.109	198.0 (180.6–199.3)	160.8 (142.6–179.6)	0.152
Serotonin (ng/ml)	35.1 (2.8–56.5)	23.8 (0.0–85.7)	0.732	29.3 (25.1–46.9)	25.3 (0.0–39.5)	0.386
Kynurenine (µmol/l)	2.4 (2.0–3.2)	2.5 (2.0–2.8)	0.788	2.4 (2.1–2.7)	2.2 (1.8–2.8)	0.390
Quinolinic acid (ng/ml)	3.3 (3.1–3.8)	3.2 (3.0–3.8)	0.207	3.1 (3.0–3.7)	3.2 (2.7–3.6)	0.512
Serotonin/tryptophan ratio	0.2 (0.0–0.4)	0.15 (0.0–0.5)	0.710	0.1 (0.1–0.2)	0.1 (0.0–0.3)	0.592
Kynurenine/tryptophan ratio	0.0 (0.0–0.1)	0.02 (0.01–0.02)	0.403	0.0 (0.0–0.0)	0.0 (0.0–0.0)	0.269
Serotonin/kynurenine ratio	6.0 (1.7–23.1)	10.9 (0.0–32.1)	0.753	14.2 (10.5–17.1)	10.9 (0.0–26.6)	0.483

Data presented as medians (interquartile ranges)

*BDI* Beck's Depression Inventory, *BMI* body mass index, *BASDAI* Bath Ankylosing Spondylitis Disease Activity Index, *VAS* visual analog scale, *ESR* erythrocyte sedimentation rate, *hsCRP* high sensitivity C-reactive protein, *WBC* white blood cells, *N/L ratio* neutrophils to lymphocytes ratio, *RBC* red blood cells, *Hgb* hemoglobin, *PLT* platelets

## Discussion

As expected, the results of our study showed the altered tryptophan metabolism with the predominance of the kynurenine pathway in patients with ankylosing spondylitis compared to the control group. Although the sex distribution between the study group and the control group was not identical, and this may have an effect on inflammation, tryptophan metabolism, and depression (Casimir et al. 2010; Songtachalert et al. 2018; Knight et al. 2020), the results of our study confirmed the reports from the literature (Eryavuz Onmaz et al. 2021; Klavdianou et al. 2016). The tryptophan kynurenine pathway has been considered as an important player in inflammation. A small amount of tryptophan is converted to serotonin and melatonin, while approximately 95% is metabolized to kynurenine via indoleamine 2,3-dioxygenase 1 and 2 (IDO-1, IDO-2) or tryptophan 2,3-dioxygenase (TDO2). Under normal conditions, tryptophan is metabolized mainly by TDO2, while IDO plays a minor role (Ye et al. 2019). However, in many diseases characterized by chronic inflammation, such as autoimmune disease, IDO-mediated tryptophan metabolism is strongly activated (Åkesson et al. 2018). Overproduction of pro-inflammatory cytokines such as TNFα, IL-6, IL-17, IL-23 contributes to altered tryptophan metabolism (Müller 2014). Thus, we expected that effective treatment of inflammation in patients with ankylosing arthritis, by directly blocking TNFα, would result in an improved balance in tryptophan metabolism.

Surprisingly, we found no significant changes in serum levels of tryptophan and its metabolites in AS patients treated with TNFα inhibitors, despite clinical improvement. Moreover, the components of tryptophan metabolism did not correlate significantly with the laboratory markers of inflammation. Taking into account that increased tryptophan metabolism toward kynurenine could be caused by elevated TNF-α levels (O'Connor et al. 2009; El-Bakly and Hasanin 2014; Lorkiewicz and Waszkiewicz 2021; Milaneschi et al. 2021; Schiller et al. 1992), the results of our study could be quite surprising. However, these postulated mechanisms of indoleamine 2,3-dioxygenase (IDO) activation by TNFα, leading to the predominance of the kynurenine pathway, have been studied in preclinical research (O'Connor et al. 2009; El-Bakly and Hasanin 2014). Similar observations in human studies concerned different groups of patients than in our study—ex. infection (Lorkiewicz and Waszkiewicz 2021) and cancer (Schiller et al. 1992). Whereas, the results of our study are partly consistent with previous reports on a group of patients with ankylosing spondylitis, in which effective therapy with TNFα inhibitors resulted in a decrease in kynurenine/tryptophan ratio, although the effect of treatment on other metabolites appeared to be limited (Eryavuz Onmaz et al. 2021) or even serotonin levels decreased during biological treatment (Klavdianou et al. 2016).

A partial explanation for our observation may be that many factors influence the pathways of tryptophan metabolism (Correia and Vale 2022). Anti-TNFα therapy is a very effective therapy for rheumatic diseases, but does not modulate all inflammatory pathways. Both TNFα and interferon-gamma (IFNγ) represent potent pro-inflammatory cytokines, known to be able to interfere with tryptophan metabolism pathways (Werner-Felmayer et al. 1989). It seems probable that TNFα inhibitors could modulate immune function without downregulating biochemical pathways that are under the control of IFNγ, whereas other inflammatory cascades are significantly suppressed. TNFα inhibitors might exert their beneficial effects by modulation of primarily Th2-type immune response independent from any effect on pathways modulated by IFNγ. But also in this case, there should still exist an interaction between Th2-type and Th1-type immunity, which should indirectly reduce the activation of the kynurenine pathway (Schuerwegh et al. 2003). So, it does not completely explain our results; therefore, these mechanisms are probably more complex.

Ankylosing spondylitis is a disease characterized by excessive bone formation in the form of ectopic ossification. The molecular pathways involved in new bone formation in ankylosing spondylitis are complex and not fully understood (Klavdianou et al. 2022). The TNFα inhibitors can increase bone mass density (BMD) in patients with AS (Kang et al. 2013). At the same time, serum serotonin levels in patients with high bone mass syndrome associated with Lrp5 mutation were measured lower than healthy controls (Frost et al. 2010) and inverse correlation of serotonin levels with BMD was identified in these patients (Frost et al. 2011). Thus, serotonin may be also involved in new bone formation in AS, which may contribute to the paradoxical effect of TNF inhibitors on tryptophan metabolite concentrations (Klavdianou et al. 2016, 2022). However, this hypothesis also does not completely explain the results of our study.

Summarizing, we report herein that treatment with TNFα inhibitors in ankylosing spondylitis patients had no effect on tryptophan metabolism pathways; however, we cannot clearly identify the mechanisms responsible for these observations. To the best of our knowledge, this is the first study to investigate the effect of TNFα inhibitors on the balance between the kynurenine and the serotonin pathways in patients with ankylosing spondylitis. Potential limitation of our study is the small size of our cohort. Our study has also some other disadvantages: like lack of measurement of kynurenine pathway enzyme activities and other inflammatory markers. An additional limitation is the unequal gender distribution in the study group and the control group. Due to the above limitations, our results can only be regarded as preliminary. To investigate this observation, long-term studies examining possible downstream pathways modulated by TNFα inhibitors are needed.

## Conclusion

The altered tryptophan metabolism with the predominance of the kynurenine pathway in patients with ankylosing spondylitis, compared to the control group, was demonstrated. Surprisingly, there was no significant changes in serum levels of tryptophan and its metabolites in patients with ankylosing spondylitis treated with TNFα inhibitors, despite clinical improvement. Moreover, the components of tryptophan metabolism did not correlate significantly with the clinical activity of the ankylosing spondylitis, depression nor laboratory markers of inflammation. Probably some other factors influence the pathways of tryptophan metabolism in patients with ankylosing spondylitis.

## Data Availability

All data generated or analyzed during this study are included in this published article.
